# Broken Trust. Confidence Gaps and Distrust in Latin America

**DOI:** 10.1007/s11205-021-02796-3

**Published:** 2021-09-10

**Authors:** Paolo Parra Saiani, Enrico Ivaldi, Andrea Ciacci, Lucia Di Stefano

**Affiliations:** 1 Department of Political Sciences, University of Genoa, Genoa, Italy; 2 Department of Political Sciences, C.I.E.L.I., the Italian Centre of Excellence on Logistics Transports and Infrastructures, University of Genoa, Genoa, Italy; 3 Centro de Investigaciones en Econometría – CIE University of Buenos Aires, Buenos Aires, Argentina; 4 Department of Economics, Center for Security, Risk and Vulnerability, University of Genoa, Genoa, Italy

**Keywords:** Institutional trust, Government, Latin America, Aggregative method, Temporal index

## Abstract

Latin American societies show lower levels of political trust when compared to other regions of the world. The lack of trust in institutions can led to ineffective management of public affairs, social crises, lack of transparency, economic problems and even difficulties in countering pandemics. The objective of this work is to build an index (LADI) that provides a measure of the level of perceived distrust in the institutions of the different Latin American countries and its variations over the period from 2008 to 2018. The data used for this analysis are of a subjective nature and come from the series of surveys provided by *Latinobarómetro*. To develop the analysis, we have used a quantitative approach of a partially non-compensatory aggregative type, known as Adjusted Mazziotta and Pareto Index. The results show a generalized increase of distrust in the years 2017 and 2018 for several Latin American countries. On the other hand, in countries where the rule of law is more consolidated, a best perception of the functioning of democracy emerges.

## Introduction

In recent years, there has been a renewed interest in the so-called “confidence gaps”, which would constitute threats to the legitimacy of democratic institutions and obstacles to economic growth. Trust has been considered as a central dimension of social capital, a necessary condition of social integration, economic efficiency, and democratic stability (Arrow, 1972; Coleman, 1988; Putnam, 1993, 2000; Fukuyama, 1995; Newton, 1997); trust can be defined as the set of socially learned expectations that people have regarding to other individuals, to organizations and institutions, and to the moral and social order. The concept of trust is related to expectations that have a positive value for the social actor and which are formulated in conditions of uncertainty. This applies both when the receiver of such expectations is the natural and social organisation as a whole or its individual institutional and collective expressions (*systemic* or *impersonal trust*), and when the receiver is made up of individual actors (*personal* or *interpersonal trust*) (Rotter, 1980; Misztal, 1996; Frederiksen, 2014). While the concept of trust is referred to both kinds of relations, it is necessary to differentiate them: if intersubjective trust has to do with the contingency associated with the agency of the other, the institutional trust deals with performance and reliability (Luhmann, 1988; Seligman, 1997; Hardin, 2002).

Compared to interpersonal trust, the mechanisms of systemic and institutional trust are less studied. Economic crisis often aggravates the conditions in which local governments have to manage territories, with an impact on local quality of life. The quality of life at local level can be one of the determinants of trust or distrust in the institutions: “if individuals perceive government as having a primarily negative impact on their quality of life, a reluctance to trust government is a likely outcome” (Yonk & Smith, 2018). The level of this uncertainty is the complex product of several factors: “Normally, trust is a response to good institutional performance, but it is also an essential condition for effective governance. It is the basis for compliance with the rules. Trust in political institutions not only encourages citizens to pay taxes or to support reforms that require short-term costs, in view of long-term benefits” (Mingo & Faggiano, 2020), but may save lives: as Elgar, Stefaniak and Wohl have pointed out recently, “confidence in institutional authorities and low-income inequality may facilitate public health advice […]. Specifically, vaccination rates may differ between countries as a function of income inequality and social capital. In summary, our analysis found that COVID-19 mortality relates to income inequality and specific dimensions of social capital after other cross-national differences in wealth, population size, and population age were controlled” (2020). Marlow et al. (2007), van der Weerd et al. (2011) and Ozawa and Stack (2013) reach the same conclusions.

The distance created between the actor and the system favours the perception of an absence of alternatives (Simmel, 1900; Luhmann, 1988). Institutional trust is widespread in many countries and represents the feeling of de-legitimization that is considerably involving a wide range of social and political institutions: institutions are then seen as far from citizens’ needs and expectations. Like in Wright (1976) or Hart (1978), distrust is not always the product of ignorance and unrealism or a reaction to mass society: some dose of distrust in institutions “may be a healthy sign of citizens’ aloofness from a sphere of social life on which they have little control” (Moisés, 2006; see also Pettit, 1998; Sztompka, 1999; Warren, 2001). The prevalence of deprived and powerless social groups among those who express political distrust shows that it is the result of rational and realistic attitudes. The heterogeneity of these groups, their fatalism, and their willingness to pragmatically accept the status quo mean that the negative attitude towards the political system is not translated into effective opposition. The more complex societies are, the more generalised is the need for both interpersonal and institutional trust, since there is a significant positive correlation between trust in oneself and others, and trust in institutions (Erikson, 1968; Lipset & Schneider, 1983; Newton & Norris, 2000; Allum et al., 2010).

As Warren argues (1999), there is a strong connection between democracy and trust, saying that they are distinct but complementary ways of making collective decisions and organizing collective actions. Trusting institutions is not the same thing as trusting individuals. In addition, institutions are like bicycles, because they cannot be the objects of genuine trust, but only the objects of empirical or theoretical knowledge and beliefs (Ibidem). Remembering also that institutional trust can be seen as a result of individual’s limited information, Offe (1996) suggests strategies that can address the deficit of trust in institutions. Trust can increase if institutions develop norms of truth-telling, solidarity, promise-keeping, but also develop the habits and dispositions of extending trust to strangers by increasing citizen involvement in associational life.

The aim of this paper is to construct an index, called Latin American Distrust Index (LADI) that provides a measure of the perceived distrust level toward the institutions of the different Latin American countries. LADI enables one to capture the variations over the time period 2008–2018. To pursue the aims, the subjective data provided by Latinobarómetro surveys are used. The analysis is based on the development and application of a partially non-compensatory aggregative quantitative method, known as Adjusted Mazziotta and Pareto Index (AMPI).

This paper intends to contribute to literature in several ways. Mainly, it expands the extent knowledge in the field of the trust phenomenon towards the Latin American countries institutions from a political-sociological perspective. Second, it shows how the quantitative methods of analysis, and in this specific case the AMPI method, can be employed in different fields of research effectively. Third, it proposes a cross-contribution dealing with a delicate issue, i.e. trust in institutions. Such a theme is prone to be deepened from different analytical perspective in further work in different disciplines, given the pervasive effects that high or low levels of trust can produce in the broad frame of a country-system.

## Data

The data used in this analysis come from *Latinobarómetro* surveys.^1^ We have adopted the sample of Latin American countries to acquire the perceived level of distrust towards institutions in the Latin American Region. Data are of a subjective nature. They are expressed as a percentage frequency and represent the percentage of respondents who are satisfied and confident in the functioning of the different institutions.^2^ The data represent a historical series as they refer sequentially to several different years. In the specific, data refer to the period from 2008 to 2018. For the two-year periods 2011–12 and 2013–14 are available only two surveys, one for each two-year period. Surveys involved approximately 20,000 interviews each year, constituting—at least for the latest waves—representative samples of 100% of the population of each country. As a result, Latinobarómetro (2018a) states that surveys are representative of the region’s population on the basis of stratification criteria.^3^

The employed dataset is constituted by 5 indicators, described in Table 1. The aggregation process has been developed by considering these elementary indicators.

**Table 1:** Indicators description

Indicator	Scale
Satisfaction with democracy	1. Very satisfied 2. Rather satisfied 3. Not very satisfied 4. Not at all satisfied -1. Don´t know -2. No answer/refused -3. Not applicable -4. Not asked
Confidence in National Congress/in Parliament	1. A lot of confidence 2. Some confidence 3. Little confidence 4. No confidence at all -1. Don´t know -2.- No answer/refused -4.- Not asked
Confidence in the Judiciary	1. A lot of confidence 2. Some confidence 3. Little confidence 4. No confidence at all -1. Don´t know -2.- No answer/refused -4.- Not asked
Confidence in the Police	1. A lot of confidence 2. Some confidence 3. Little confidence 4. No confidence at all -1. Don´t know -2.- No answer/refused -4.- Not asked
Confidence in the Government	1. A lot of confidence 2. Some confidence 3. Little confidence 4. No confidence at all -1. Don´t know -2.- No answer/refused -4.- Not asked

*Source:* Latinobarómetro (2018c)

In order to evaluate the level of distrust for each Latin American Country, it has been built a temporal index, indicated by using the acronym LADI. The direction (sign) of the index is negative. In this sense, higher LADI values correspond to higher levels of distrust.

Some indicators may be positively correlated with the phenomenon to be measured (positive polarity), whereas others may be negatively correlated with it (negative polarity); since all the indicators must have positive polarity, we reverse those with negative polarity by using the transformation proposed by Mazziotta & Pareto (2018) for AMPI.

## Methods

Starting from a formative approach (Maggino, 2017), the aim is to construct an index to evaluate the level of trust that characterizes the different Latin American countries. Adopting a formative (or causal) approach means that the elementary indicators are assumed to be cause the latent variable, instead of being caused by it. In these terms, some variations in the formative indicators change the value of the latent variable (Blalock, 1968).

The choice of the right measurement perspective should be theory-driven (Diamantopoulos & Siguaw, 2006). In this regard, it is necessary to specify in advance what relationships characterize the theoretical framework of reference (Edwards & Bagozzi, 2000). The formative approach choice has been previously evaluated on the basis of the nature proper of the phenomenon to be studied. Since it has been clarified that the elementary indicators employed in this study are cause of higher/lower levels of political trust, and not vice versa, the formative solution is the most suitable (Diamantopoulos & Winklhofer, 2001).

One of the most common problems affecting the construction of indexes is the compensation resulting from the aggregation of uneven indicators, namely composition-through-compensation fallacy (Alaimo & Maggino, 2020). Specifically, it refers to those situations where an index can produce the same values for different situations (Ibidem). In order to better explain what compensation refers to, it is necessary to clarify the meaning of “substitutable” and “non-substitutable” indicators. The former indicates the possibility to compensate a “deficit” in one indicator by a “surplus” in another (Mazziotta & Pareto, 2017, 174) and the latter refers to the opposite situation. Compensation is often not allowed in the case of a formative approaches-based measurement, therefore indicators with different meanings don’t allow the compensation (non-substitutability), since such a mathematical operation would imply the violation of their initial semantic character (Maggino, 2017). This is the reason why when dealing with the formative approach, it is important to ensure the partial non-compensability of the aggregation method.

A further limitation of most of the methods used to construct indicators is the impossibility of producing measures that are comparable over time (Mazziotta & Pareto, 2018). The problem that arises can be summarized as follows: how can an index be produced whose values represent the evolution of a phenomenon over a given period of time?

The solution offered by Mazziotta and Pareto (2017) proposes the construction of an index aimed at the analysis of historical series and able, at the same time, to partially limit the compensatory effect deriving from the aggregation. This approach is called Adjusted Mazziotta and Pareto Index (AMPI), a variant of the Mazziotta and Pareto Index (MPI) (Mazziotta & Pareto, 2017, 2016). Many applications of AMPI have been proposed (Alaimo et al., 2020a; Alaimo & Maggino, 2020; Ciacci et al., 2020; D’Urso et al., 2020; Ivaldi et al., 2020a; Ivaldi & Ciacci, 2020), because it can be employed to conduct analysis in several research fields. Another approach to building indicators comparable over time is the so-called “stacking deprivation” (Norman, 2010; Landi et al., 2018). Stacking deprivation produces more compensatory results than those produced by AMPI (Mazziotta & Pareto, 2019).

There are also non-aggregative solutions to compare set of indicators over time (Alaimo et al., 2020b). According to this approach, a method for the analysis of time series is represented by temporal Poset (Alaimo et al., 2020a). However, many contributions have been developed on an aggregative analytical system, well established in the literature (Ciacci et al., 2021; Ciacci & Tagliafico, 2020; Ivaldi et al., 2020b; Mazziotta & Pareto, 2020; Penco et al., 2020; Ivaldi et al., 2018). A strength of the AMPI method is represented by the application of a penalty function to the statistical units. The amount of the penalty is related to the statistical units’ variable tendency to assume even or uneven values of its indicators. The effect of the penalty is to limit the compensation deriving from the aggregation of indicators characterized by not aligned values. For these reasons, the AMPI method is the best to use.

AMPI procedure calculation is a step-by-step process (Mazziotta & Pareto, 2018), i.e., a process developed in more phases. They are identifiable in a min–max normalization and aggregation. For the formalization of the AMPI formulas, see Mazziotta and Pareto (2018, from 968 to 970 pages).

So it is necessary to adopt the “negative” form of the AMPI index, since increasing values of the index correspond to negative variations of the phenomenon, i.e., distrust.

The AMPI procedure was important to calculate LADI index. The work on the elementary indicators (Table 1) was done without building intermediate dimensions. Given the negative polarity of all the indicators, the correct interpretation of the LADI index suggests that the higher LADI values correspond to higher levels of distrust.

## Results

Table 2 shows the scores established by the different Latin American countries with regard to the distrust over time. Starting from the first year of detection (LADI-08), the index shows high levels of perceived distrust in Peru (127.7), Panama (114.7) and Guatemala (114.48). On the other hand, a high trust in institutions is placed in Uruguay (73.75), El Salvador (88.54) and Chile (91.03).

**Table 2:** LADI results

Country	LADI-08	LADI-09	LADI-10	LADI-11-12	LADI-13-14	LADI-15	LADI-16	LADI-17	LADI-18
Argentina	106.29	113.76	99.50	89.14	95.02	96.31	90.96	92.98	92.79
Bolivia	106.58	101.46	110.63	108.84	106.76	96.96	104.80	96.62	95.92
Brazil	94.34	97.20	87.85	97.76	99.12	111.13	114.91	116.56	111.73
Chile	91.03	86.53	85.05	104.78	105.85	97.13	102.88	97.40	80.62
Colombia	92.27	94.66	97.85	102.02	105.34	108.91	108.48	109.94	97.08
Costa Rica	101.68	86.52	88.74	98.75	103.74	96.52	99.17	89.21	75.15
Dom. Rep	97.76	98.49	111.21	114.72	92.00	87.51	84.69	103.14	107.96
Ecuador	99.07	108.28	103.13	81.51	73.22	80.62	82.02	80.54	90.01
El Salvador	88.54	88.72	104.16	95.00	103.16	116.75	114.36	113.82	122.72
Guatemala	114.48	115.99	121.98	125.38	114.73	110.12	107.23	108.18	113.56
Honduras	108.97	113.02	100.90	112.97	121.71	105.81	112.46	110.91	107.17
Mexico	108.97	113.84	113.46	113.42	109.54	113.35	96.29	112.72	105.74
Nicaragua	103.41	115.02	108.96	103.89	90.46	90.25	94.65	84.12	118.09
Panama	114.70	89.36	88.63	86.84	99.32	109.93	107.50	101.88	99.16
Paraguay	97.21	101.95	105.92	104.92	112.20	104.16	110.38	119.13	90.64
Peru	123.70	122.38	122.04	113.46	120.58	114.30	112.56	115.58	114.91
Uruguay	73.75	67.17	66.62	65.48	72.50	67.20	70.53	68.04	70.26
Venezuela	93.14	100.69	93.42	92.12	91.31	107.26	110.31	97.66	117.91

In 2009, there were some changes in the LADI-09 ranking compared to the previous year. Peru (122.38) and Guatemala (115.99) confirmed their position as the countries with the highest levels of distrust; Nicaragua, with a LADI-09 coefficient equal to 115.02, shows a clear increase compared to 2008 (+ 11.61). On the other hand, trust in Panama (89.36) has risen convincingly, and low levels of distrust of institutions El Salvador (88.72), Chile (86.53), Costa Rica (86.52) and Uruguay (67.17) are confirmed.

In 2010, the perceptions in Peru (122.04), Guatemala (121.98) and Mexico (113.46) remained almost unchanged, while the distrust grew in Dominican Republic (111.21), Bolivia (110.63) and El Salvador (104.16). On the other hand, the main decreases in the value of LADI-10 can be seen in the cases of Honduras (100.90), Argentina (99.50) and Brazil (87.85).

LADI-11-12 shows some more pronounced variations than in the previous three years. While the countries with the highest level of distrust remain the same (Guatemala (125.38), Dominican Republic (114.72) and Peru (113.46)), there is again an increase in distrust in Honduras (112.97), after the sharp decrease in LADI-10. Chile (104.78) leaps to the middle of the ranking, with a strongly growing level of distrust. Increases of distrust also emerge in Costa Rica (98.75) and Brazil (97.76), while trust in Argentina is strengthened (89.14). Ecuador is the country that more than all the others show an increase in trust (81.51, + 21.63 compared to LADI-10).

The surveys for the years 2013–14 (LADI-13-14) and 2015 (LADI-15) show the greatest instability in the perception of trust/distrust in Latin America. Specifically, looking at LADI-13-14, Honduras (121.71) and Peru (120.58) show a strengthening of the feeling of distrust towards institutions, as well as Panama (99.32), which is settling at a lower overall level of distrust. The most noticeable change in trust can be found in Dom. Rep. (92.00, − 22.72 on LADI-11-12), as well as an apparent strengthening of trust in institutions emerges in Nicaragua (90.46, − 13.43).

LADI-15 shows variations among the countries with the highest levels of distrust. El Salvador (116.75) acquires the little hoped-for first position for the highest level of scepticism towards democracy and its institutions. There is further accentuated growth for Brazil (111.13), the fourth country with the highest level of distrust, Panama (109.93) and Venezuela (107.26).

According to the results of LADI-16, there are no particular changes in 2016. The only change worthy of note is recorded in Mexico (96.29, − 17.06 compared to the score resulting from LADI-15).

LADI-17 shows a situation that is deeply changed, if compared with previous years, due to changes (even small ones) that have occurred incrementally over the years. The countries with the greatest perceived distrust are Paraguay (119.13), Brazil (116.56), Peru (115.58), El Salvador (113.82), Mexico (112.72). Among the countries in which respondents express the highest trust in their institutions are Argentina (92.98), Costa Rica (89.21), Nicaragua (84.12), Ecuador (80.54) and Uruguay (68.04).

LADI-18 offers a changed picture from the one initially analyzed with LADI-08. On this basis, 2018 can be identified as a year of rupture. In fact, LADI-18 shows a high variability compared to the previous year, with sudden changes in population opinion in several countries. El Salvador ranks first for its high distrust (122.72), which grew further between 2017 and 2018 (+ 8.89). In Nicaragua (118.09) and Venezuela (117.91) there were real upheavals, with an increase in distrust reaching a peak of + 33.97 for Nicaragua and + 20.26 for Venezuela. On the contrary, increases in trust would emerge convincingly in Colombia (97.08), Chile (80.62), Costa Rica (75.15) and, above all, in Paraguay (90.64, − 28.49 compared to LADI-17).

**Fig. 1:**
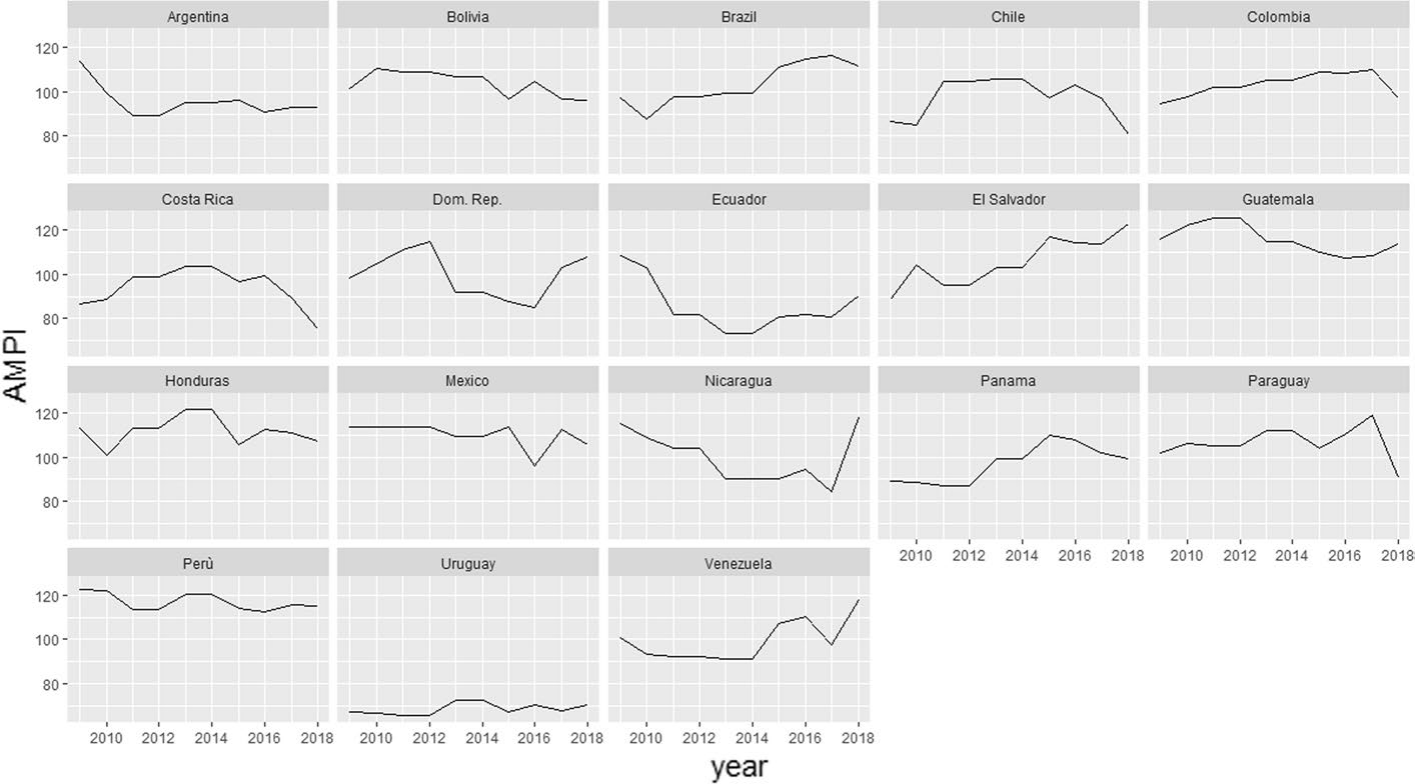
Graphic representation of the time-series

Figure 1 shows the fluctuation in levels of distrust during the years for each Latin American country. With 100 being the average reference value for the LADI index, only Uruguay has levels of distrust below the average threshold for all the years observed. Argentina, starting from 2010, falls below the average threshold for all the following years. A similar result is also established by Ecuador, which reaches its minimum in the years 2013–14. Costa Rica, whose trend is mainly below the average threshold, exceeds 100 on only two occasions: 2008 and 2013–14.

On the contrary, there are some cases of prolonged distrust towards the institutions of their country: El Salvador constantly follows a trajectory of growing distrust, starting from the 2008 minimum to the 2018 maximum. Guatemala, Honduras, and Peru never fall below the threshold level, showing a deep-rooted distrust of their institutional system.

## Discussion and conclusions

This paper wants to analyse the level of trust in political institutions in Latin American countries and its variations over time, from 2008 to 2018. To do this, it has been adopted a partially non-compensatory aggregative analysis method known as AMPI, obtaining an index of distrust (LADI) for each year of detection. From the values of the indices, in most cases, there are significant variations over a few years or from one year to the next. The picture appears mostly unstable and susceptible to shocks that can be linked to the political sphere of influence.

The years 2017 and 2018 were the years that presented the deepest inflections of trust in democracy and institutions. The situation appears to be growing sharply in the year 2017 for Brazil and Mexico. This sentiment led in 2018 to the election of two presidents with a radical orientation (Hunter & Power, 2019), candidates with a different profile from that typically offered by the political system in the two countries. The high levels of distrust established in Honduras, and which remained almost constant throughout the decade, are to a large extent related to high crime rates (Consejo Ciudadano para la Seguridad Publica y Justicia Penal, 2011) and opaque elections, as well as a regime that resorted to alleged human rights violations (IACHR, 2019) to suppress political dissent; but it may be relevant the initial enthusiasm with which Latin American citizens received democracies during the transition, then reduced because the established democratic systems have not been able to meet the basic needs of certain social groups (Catterberg & Moreno, 2006: 33; Delgado Sotillos, 2015, 140). Deep economic inequalities and uncertainties underlie the lack of trust in Venezuela; in addition to this, Venezuela has been torn by deep political crises, that can lead to threats to the social cohesion of the country (Morselli et al., 2020). The source of the high level of distrust measured in Peru is the corruption (Goldstein & Drybread, 2018). In Nicaragua, where the very-controversial regime of Daniel Ortega is in force (Thaler, 2017), distrust has reappeared with a sharp increase from 2017 to 2018. By contrast, prosperous countries such as Uruguay, Costa Rica and Chile, where the rule of law is relatively well established, are the countries most satisfied with the way democracy (Kurtenbach & Nolte, 2017) works.

As many will remember, Chile has been the scene of protests erupted in 2019–2020, and the fact that thousands and thousands of people in the streets rallied against government may seem contradictory. A possible explanation of this (apparent?) inconsistency, is that ‘trust’ is a polysemic concept, recalling various meanings as well as different cognitive objects, thus mixing trust in persons holding a position and the position itself (Pitrone, 2009): Chileans may still have high trust in institutions, while disagreeing with government. Another possible explanation which requires further investigation, is that “in countries where trust in institutions is low, citizens often express their consent for political candidates—not rarely populists and radicals—who promise immediate benefits and quick solutions to complex problems” (Mingo & Faggiano, 2020; see also Kriesi et al., 2012; Del Tronco, 2013; Olivera, 2014; Kriesi & Pappas, 2015; Morlino & Quaranta, 2016; Muro & Vidal, 2016; Morlino & Raniolo, 2017; Arpino & Obydenkova, 2019). In this case, protesters asked for a new constitution, not exactly a “quick solution” nor a solution providing “immediate benefits”, so indirectly confirming that a high level of trust in institutions is plausible even in times of political crisis and unrests if configured as political participation and civic engagement (Kanacri & Jiménez-Moya, 2017). As Lewis and Weigert pointed out, “An informed democratic citizenry, then, retains both adequate distrust of individual politicians, and an abiding trust in the political system itself” (1985).

However, the unequal trends between countries with respect to the citizen assessment of Latin American democracies and determining their causes is still a complex issue that requires analysis and specific explanatory elements in each of the countries (Delgado Sotillo, 2015, 140). As stated before, distrust is not always the product of ignorance and unrealism or a reaction to mass society, since distrust in institutions may be a rational citizens’ reaction, as the prevalence of deprived and powerless social groups among those who express political distrust shows. Historical series analysis has allowed us to measure the levels of trust within the temporal range 2008–2018 for the eighteen Latin American countries. The use of an aggregative method has made it possible to achieve unequivocal and easily communicable results (Mazziotta & Pareto, 2017). In order to test the validity of the results obtained, the recommendation is the use of a method different from the one used, which can reproduce the analysis proposed here for the purposes that characterize it. An interesting idea could lead to the use of a non-aggregative method functional to the analysis of historical series, such as the already mentioned method of the temporal Poset (Alaimo et al., 2020a).

## Footnotes

1 *Latinobarómetro* is a nongovernmental organization based in Santiago, Chile. *Latinobarómetro* administers a common questionnaire to households in Latin America with questions that involve areas such as Economy and International Trade, Integration and Regional Trading Blocks, Democracy, Politics and Institutions, Social Policies, Civic Culture, Social Capital and Social Fraud, the Environment, and Current Issues. For additional information, see www.latinobarometro.org.

2 One of the main objections to building trust indices towards institutions rests on the variability of the scores assigned by respondents to what is considered an ‘institution’, since not all institutions are considered in the same manner by respondents. Universities and churches, for example, get high scores, while Parliament and government, much lower ones (Carballo & Hermelo, 2021). In our article, we focus on the five items in Table 1, which usually report congruent scores.

3 Technical notes provide details of the sampling process and some information concerning the non-inclusion of some areas to which access is difficult; an example—not the only one—is given by some rural areas in Argentina (Latinobarómetro, 2018b).
